# Application of a nomogram to radiomics labels in the treatment prediction scheme for lumbar disc herniation

**DOI:** 10.1186/s12880-022-00778-6

**Published:** 2022-03-19

**Authors:** Gang Yu, Wenlong Yang, Jingkun Zhang, Qi Zhang, Jian Zhou, Yuan Hong, Jiaojiao Luo, Quan Shi, Zhidan Yang, Kangyu Zhang, Hong Tu

**Affiliations:** 1 Graduate School of Jiangxi, University of Traditional Chinese Medicine, Nanchang, 330004 Jiangxi China; 2 Department of Orthopedics and Traumatology, Affiliated Hospital of Jiangxi University of Chinese Medicine, Nanchang, 330004 Jiangxi China; 3 Department of Radiology, Affiliated Hospital of Jiangxi University of Chinese Medicine, Nanchang, 330004 Jiangxi China; 4 School of Computer Science, Jiangxi University of Traditional Chinese Medicine, Nanchang, 330004 Jiangxi China

**Keywords:** Radiomics, Nomogram, Lumbar Disc Herniation, Prediction Model, Treatment

## Abstract

**Objective:**

To investigate and verify the efficiency and effectiveness of a nomogram based on radiomics labels in predicting the treatment of lumbar disc herniation (LDH).

**Methods:**

By reviewing medical records that were analysed over the past three years, clinical and imaging data of 200 lumbar disc patients at the Affiliated Hospital of Jiangxi University of Traditional Chinese Medicine were obtained. The collected cases were randomly divided into a training group (n = 140) and a testing group (n = 60) at a ratio of 7:3. Two radiologists with experience in reading orthopaedics images independently segmented the ROIs. The whole intervertebral disc with the most obvious protrusion in the sagittal plane T_2_WI lumbar MRI as a mask (ROI) is sketched. The LASSO (Least Absolute Shrinkage And Selection Operator) algorithm was used to filter the features after extracting the radiomics features. The multivariate logistic regression model was used to construct a quantitative imaging Rad‑Score for the selected features with nonzero coefficients. The radiomics labels and nomogram were evaluated using the receiver operating characteristic curve (ROC) and the area under the curve (AUC). The calibration curve was used to evaluate the consistency between the nomogram prediction and the actual treatment plan. The DCA decision curve was used to evaluate the clinical applicability of the nomogram.

**Result:**

Following feature extraction, 11 radiomics features were used to construct the radiomics label for predicting the treatment plan of LDH. A nomogram was then constructed. The AUC was 0.93 (95% CI: 0.90–0.97), with a sensitivity of 89%, a specificity of 91%, a positive predictive value of 92.7%, a negative predictive value of 89.4%, and an accuracy of 91%. The calibration curve showed that there was good consistency between the prediction and the actual observation. The DCA decision curve analysis showed that the nomogram of the imaging group has great potential for clinical application when the risk threshold is between 5 and 72%.

**Conclusion:**

A nomogram based on radiomics labels has good predictive value for the treatment of LDH and can be used as a reference for clinical decision-making.

## Introduction

In recent years, the irregular life and rest patterns of modern people have led to the incidence of lumbar disc herniation (LDH) continuing to rise. Even after treatments, the disease still recurs frequently. Meanwhile, onset occurs at earlier ages and at more a serious degree, affecting daily life [1]. Lumbar disc herniation of lumbar degenerative changes refers to a variety of reasons for the development of tis disorder, including external force damage and long-term lumbar damage caused by bad habits. such as part of some or all of the rupture, and highlight the nucleus pulposus, rupture, stimulate or oppressed nerve root, horsetail nerve palsy is a clinical syndrome, is one of a common cause of low back and leg pain. Surgeries and conservative treatments are effective methods for the treatment of lumbar disc herniation. Surgical treatment can be mainly divided into traditional surgery and minimally invasive surgery. Through data analysis, some researchers believe that surgery has obvious advantages compared to conservative treatments in improving lumbar and leg pain, muscle paralysis, low quality of life and adverse reactions caused by this disease [2]. However, studies have confirmed that conservative treatments can effectively alleviate the symptoms of LDH, and thus, they are considered the first-line choice for most patients. The early efficacy of LDH is no worse than that of surgery by improving the lifestyle, physical therapy, traditional Chinese medicine treatment and routine use of drugs [3]. From the most basic forms of treatment, such as bed rest, traction, and functional exercises, to the traditional internal and external use of Chinese medicine, acupuncture and massage and therapy, the combination of traditional Chinese and Western medicine of traditional Chinese medicine preparation has included sacral canal injection, small needle knife therapy, and comprehensive treatment. There are many types of treatment, and the conservative treatment of LDH curative effect is distinct; with the progress of medicine is growing and changing [4]. In the selection of surgical treatment, doctors should follow the surgical indications of LDH: (i) LDH is clearly diagnosed and ineffective after conservative treatment, affecting daily life and work; (ii) patients with significant cauda equina syndrome are rare; and (iii) patients with severe dyskinesia caused by large disc herniation or displacement and with refractory pain are rare [5, 6]. It can be seen that surgical indications are highly subjective. This indicates that how to choose the most appropriate treatment plan has been a problem of perplexing clinicians. Improper selection is likely to result in inadequate or excessive treatment. At present, there is no quantitative method to judge the treatment plan. Radiomics [7] is a method that can convert digital medical images into high-dimensional data that can be mined. It can convert visual image information into deep features for quantitative research and reveal the information contained in the images that reflect the underlying pathophysiology. The purpose of this study was to predict surgical or conservative treatments for lumbar disc herniation using a nomogram based on radiomics labels.

## Data and methods

### Clinical data

Some of the clinical medical records and imaging data of 200 patients with lumbar disc herniation diagnosed in the Affiliated Hospital of Jiangxi University of Traditional Chinese Medicine in the past 3 years were retrospectively collected. They are randomly divided into a training group and a validation group at a ratio of 7:3. The inclusion criteria were defined as follows: (1) The patient had been clearly diagnosed with lumbar disc herniation without limitation of personal basic information; (2) Lumbar MRI was performed in our hospital, and the imaging information included at least the OSag-T_2_WI sequence and OAx-T_2_WI sequence. (3) The MSU classification was used to evaluate whether patients needed surgical treatment. The case exclusion criteria were defined as follows: (1) the image quality was poor, and it was difficult to outline the region of interest (ROI) or to extract the image omics features; (2) patients with other lumbar diseases; (3) complicated with malignant tumours; (4) people with schizophrenia or severe mental disorders; (5) severe osteoporosis; (6) vertebral body compression; and (7) previous diagnosis and treatment of LDH.

### MRI examination method

Magnetic resonance imaging scanners named Signa Hde 1.5 T and Discovery MR750 (3 T) from General Electric Company (GE) were used. After lunching the exam to be ready for the diagnosis, the spinal phasing front coil is used, and the patient wearing hearing protection is instructed not to move during the examination. The lumbar scan protocol was used during scanning. The scanning sequence and azimuth included the FSE-T_1_WI sequence in the sagittal plane, the FSE-T_2_WI sequence in the sagittal plane, the FSE-T_2_WI sequence in the sagittal plane, and the FSE-T_2_WI sequence in the transverse plane. Due to the possibility of disc-bone overlap in cross-sectional T_2_WI sequences, which affects image representativeness, only sagittal T_2_WI sequences were used in this study. The main parameters included TR 2000 ms, TE 120 ms, layer thickness 4 mm, layer spacing 0.5 mm, matrix 320*224, average acquisition times twice, and field of view 320 mm*320 mm.

### Image segmentation and feature extraction

#### Segmentation of ROI

Two radiologists who have many years of experience in reading orthopaedics films independently use ITK-SNAP (v3.6.0, http://www.itksnap.org/) software [8], marking the most prominent intervertebral discs in the sagittal T_2_WI sequence of lumbar MRI by multiple layers and synthesizing the region of interest (ROI).

#### Feature extraction

The Pyradiomics package (v3.0.1, http://pyradiomics.readthedocs.io/) of the open-source Python (v3.8.5) software was used to complete the feature extraction and screening of radiomics. The image preprocessing includes resampling into 3 × 3 × 3 isotropic voxels, extracting the original image, log and wavelet features, and normalizing and discretizing at the same time (BinWidth: 5). The obtained data are analysed. First, the consistency test between observers was carried out to calculate the interclass correlation coefficient (ICC) between the features extracted from the ROI drawn by two radiologists to evaluate the repeatability of the radiomics features drawn by two radiologists. When the ICC value is larger than 0.75, the consistency of feature extraction is good [9].

#### Establishment and evaluation of radiomics labels

The least absolute shrinkage and selection operator (LASSO) was used for feature selection. The parameter lambda (λ) of the Lasso regression model was selected by cross-validation, and λ with the smallest model error was selected to retain the feature when the coefficient was not equal to zero. The multivariate logistic regression model was used to construct quantitative radiomics labels for the selected features with nonzero coefficients. A weighted linear combination of the coefficients of nonzero coefficient features was used to obtain the radiomics label score (Rad‑Score) for each patient. The receiver operating characteristic (ROC) curve [10] was used to evaluate the efficacy of the radiomics label in predicting the treatment plan of lumbar disc herniation. The area under the curve (AUC) [11], sensitivity, specificity and accuracy were calculated.

#### Establishment and evaluation of a nomogram based on radiomics labels

Patient age, sex, occupation, family history, bedplate characteristics, smoking habits, exercise and BMI were recorded. MSU evaluation results were used as dependent variables to screen out high-risk factors through univariate and multivariate logistic regression. The R statistical software package was used to establish and evaluate the consistency between the prediction of the radiomics nomogram and the actual choice of treatment plan. The Hosmer–Lemeshow test was used to analyse the degree of fit of the nomogram [12]. The Harrell consistency index (C-index) is measured to quantify the discriminative power of radiomics [13]. To evaluate the clinical application of the nomogram, we used decision curve analysis (DCA) to calculate the net benefit under different threshold probabilities. The net benefit is defined as the proportion of true positives minus the proportion of false-positives plus the relative harm of false and false negative results [14].

#### Statistical analysis

R statistical software (v4.1.0, http://www.rproject.org/) was used for statistical analysis. Count data are expressed as frequencies. The chi-square test or Fisher's exact probability method was used for comparisons between the training and testing groups. The W test was used to verify whether the measurement data followed a normal distribution. If not, ${\overline{\text{x}}} \pm {\text{s}}$ is used to represent the measurement data. The independent sample t test was used for comparisons between the two groups. If the data did not follow a normal distribution with the median (upper and lower quartile), the comparison between the two groups was performed using the Mann–Whitney U test. Lasso and logistic regression are modelled by the "glmnet" package. The software packages "rms" and "regplot" were used to construct the nomogram and establish the correction curve of the nomogram. The ROC curve is drawn by the package "pROC"; the C-index is calculated by the package "Hmisc"; the Hosmer–Lemeshow test is carried out in the package "ResourceSelection"; and the DCA decision curve is drawn within the package "rmda". P < 0.05 was considered statistically significant.

## Result

### Case data

A total of 200 patients diagnosed with "lumbar disc herniation" were included, and all of them visited the Affiliated Hospital of Jiangxi University of Traditional Chinese Medicine in the past three years. Among them, 100 patients were treated by the surgical method, and 100 patients were treated by the conservative method. Among them, there were 98 males and 102 females aged between 21.0 ~ 89.0 (55.2 ± 15.7) years. The collected cases are randomly divided into a training group (n = 140) and a validation group (n = 60) in a 7:3 ratio following the random sampling approach.

### Establishment and evaluation of radiomics labels

**Fig. 1:**
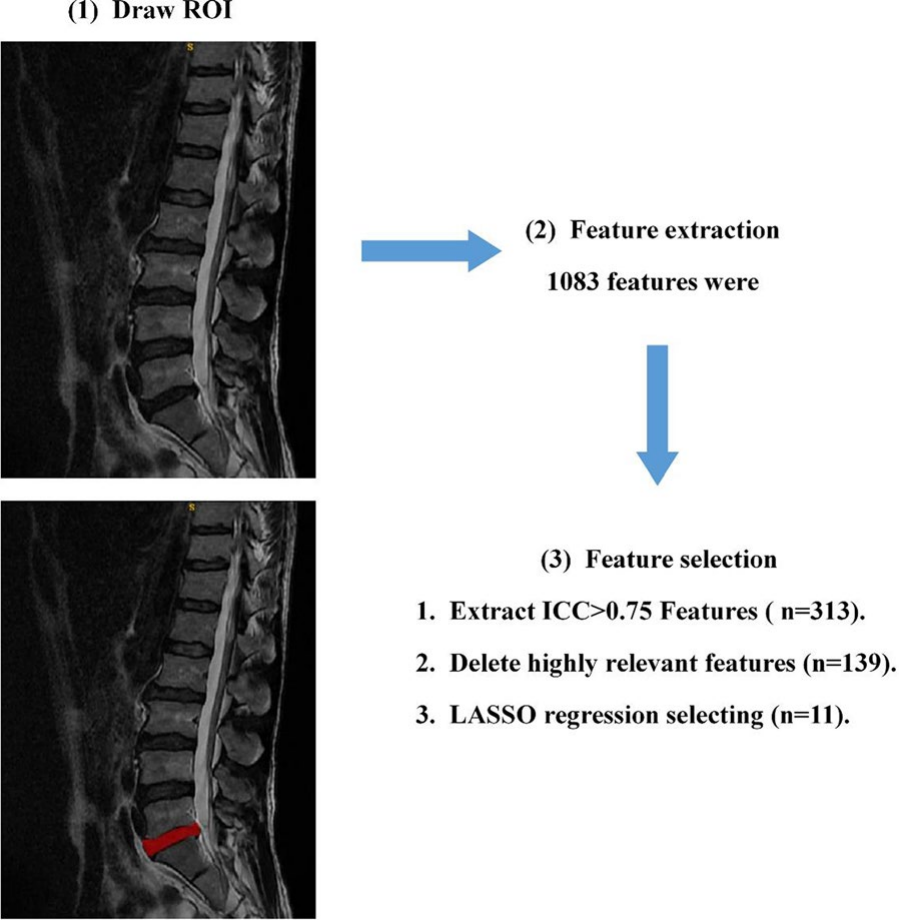
Radiomics feature extraction and screening process

**Fig. 2:**
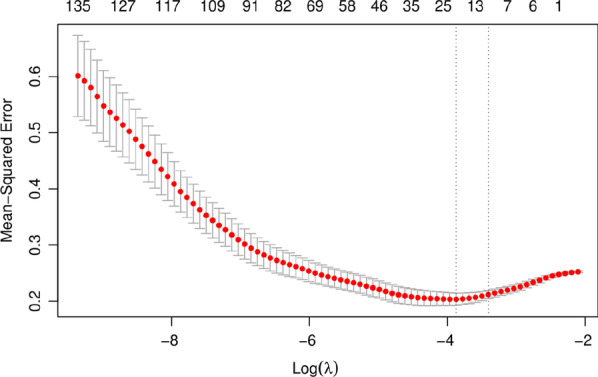
Feature selection in the least absolute shrinkage and selection operator (LASSO) model. The vertical axis is the binomial deviation, and the horizontal axis is the log(λ) value. The top number represents the number of features screened out. The smallest binomial deviation λ is the optimal value (vertical dashed line), and the optimal λ is 0.033

**Fig. 3:**
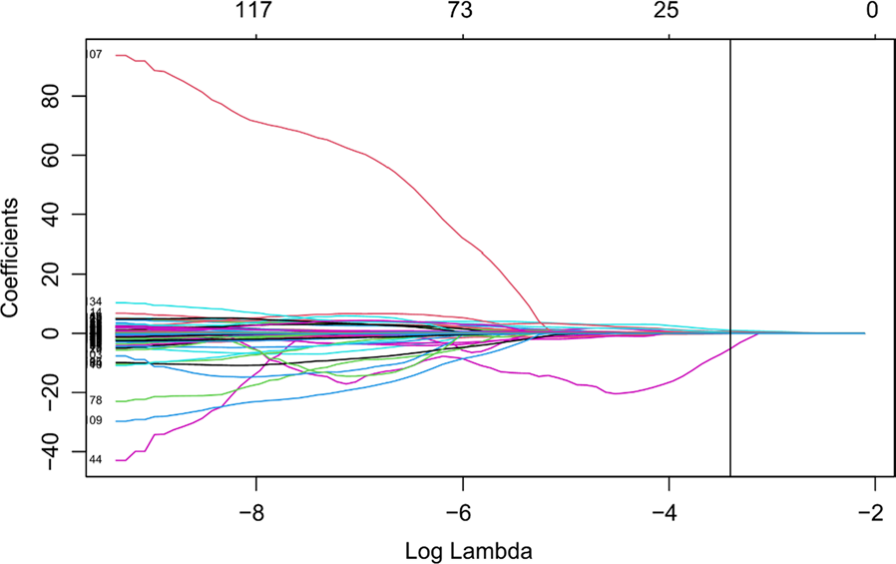
The Lasso coefficients of different features vary with the superparameter (λ value). The number above represents the number of features selected, and the black vertical line represents the 11 features with nonzero coefficients obtained

A total of 1083 features were extracted using the T_2_WI of the patient's lumbar sagittal plane (see Fig. 1), among which 313 features with ROIs greater than 0.75 were delineated by two physicians. After removing highly correlated features, 139 features remained. LASSO regression was performed for feature screening, and 11 radiomics features were obtained. Among the radiomics features, there were 5 first-order features, including the mean, interquartile range, maximum, root mean squared (RMS) error and entropy; additionally, there were 4 features of the grey level cooccurrence matrix, including correlation, inverse variance, joint energy, and informational measure of correlation (IMC1). The grey level correlation matrix feature is the dependence nonuniformity normalized (DNN), and one matrix feature of the grey size area is grey level variance (GLV). Based on the 11 selected radiomics characteristics and their coefficients, a radiomics label was constructed to predict the treatment plan of lumbar disc herniation. The best λ value obtained through calculation is 0.030, as presented in Figs. 2 and 3. A linear formulation is used to calculate the Rad-score for each patient to predict the treatment plan for lumbar disc herniation. The formula is as follows:

$$\begin{aligned} {\text{Rad-Score}}\, &= \,18.298\, + \,[0.002\, \times \,{\text{diagnostics}}\_{\text{Image}} \\ & \quad {\text{-original}}\_{\text{Mean}}\left] - \right[0.331\, \times \,\log {\text{-sigma-}}4 {\text{-}} 0 {\text{-mm-}} \\ & \quad 3{\text{D}}\_{\text{glcm}}\_{\text{Correlation}}\left] {\, + \,} \right[4.513\, \times \,\log {\text{-sigma-}} 5 {\text{-}}0 {\text{-mm-}} \\ & \quad 3D\_{\text{glcm}}\_{\text{InverseVariance}}] - \\ & \quad [22.778\, \times \,{\text{original}}\_{\text{glcm}}\_{\text{JointEnergy}}\left] {\, + \,} \right[3.133\, \times \,{\text{wavelet-}} \\ & \quad {\text{HHH}}\_{\text{gldm}}\_{\text{DependenceNonUniformityNormalized}}]\, + \,[0.009\, \times \,{\text{wave}} \\ & \quad {\text{let-HHL}}\_{\text{firstorder}}\_{\text{InterquartileRange}}\left] - \right[0.002\, \times \,{\text{wavelet-}} \\ & \quad {\text{HLL}}\_{\text{firstorder}}\_{\text{Maximum}}\left] - \right[0.085\, \times \,{\text{wavelet-}} \\ & \quad {\text{HLL}}\_{\text{firstorder}}\_{\text{RootMeanSquared}}] - [0.0008\, \times \,{\text{wavelet-}} \\ & \quad {\text{HLL}}\_{\text{glszm}}\_{\text{GrayLevelVariance}}]\, + \,[1.855\, \times \,{\text{wavelet-}} \\ & \quad {\text{LLH}}\_{\text{firstorder}}\_{\text{Entropy}}]\, + \,\left[ {1.509\, \times \,{\text{wavelet-LLL}} \_{\text{glcm}}\_{\text{Imc}}1} \right]. \\ \end{aligned}$$

**Fig. 4:**
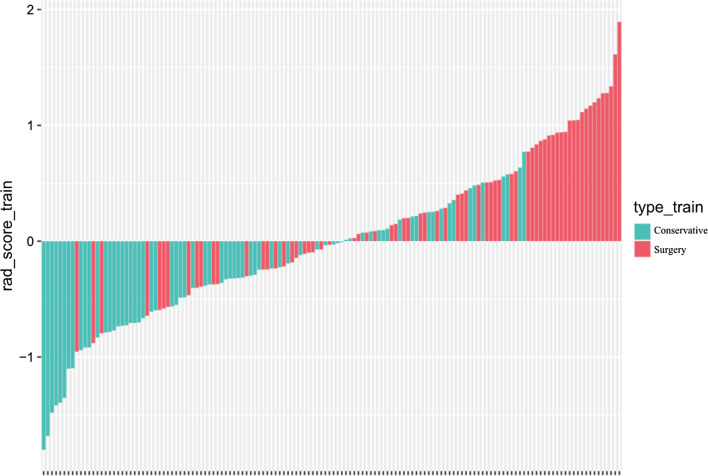
A radiomics label score for predicting treatment in the training group

**Fig. 5:**
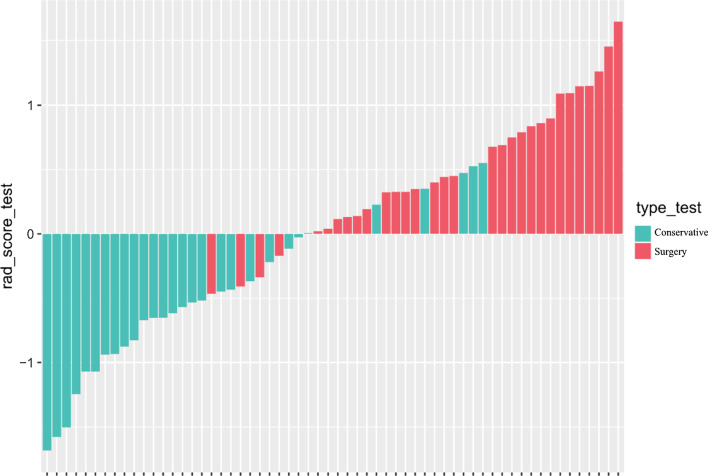
Verification of the radiomics label score of the predicted treatment plan in the group

**Fig. 6:**
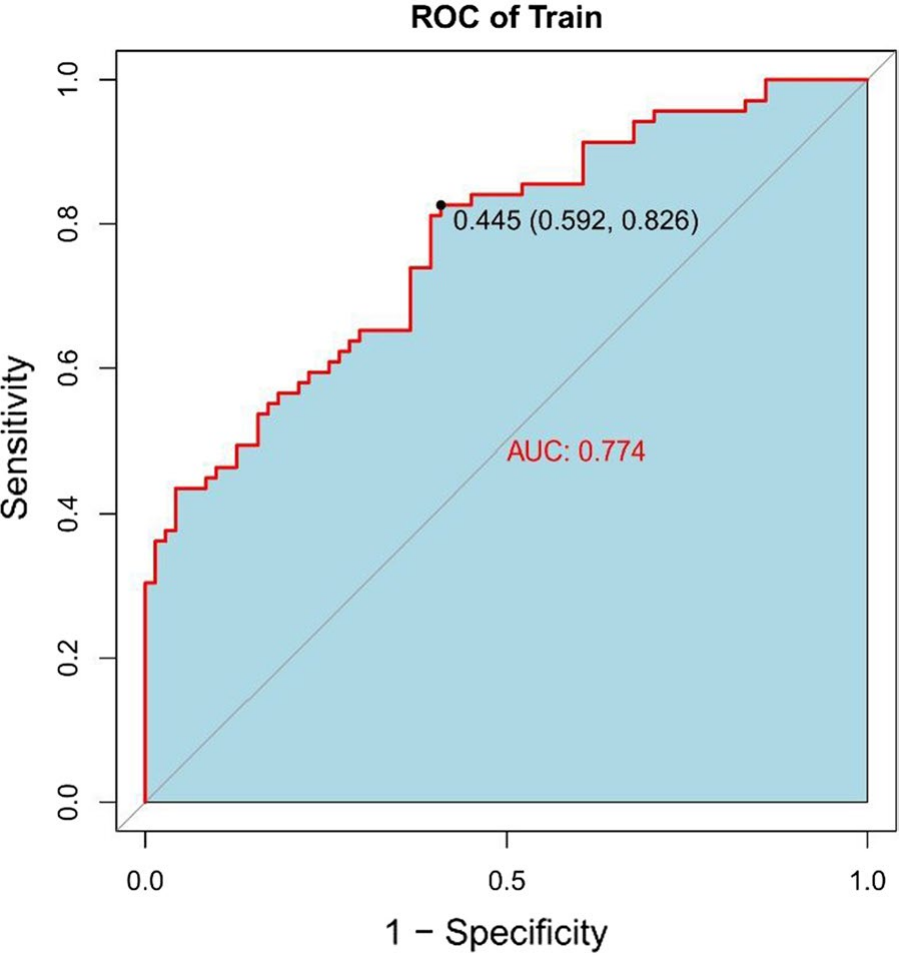
Radiomics label predicts ROC for the treatment of lumbar disc herniation of patients in the training group

**Fig. 7:**
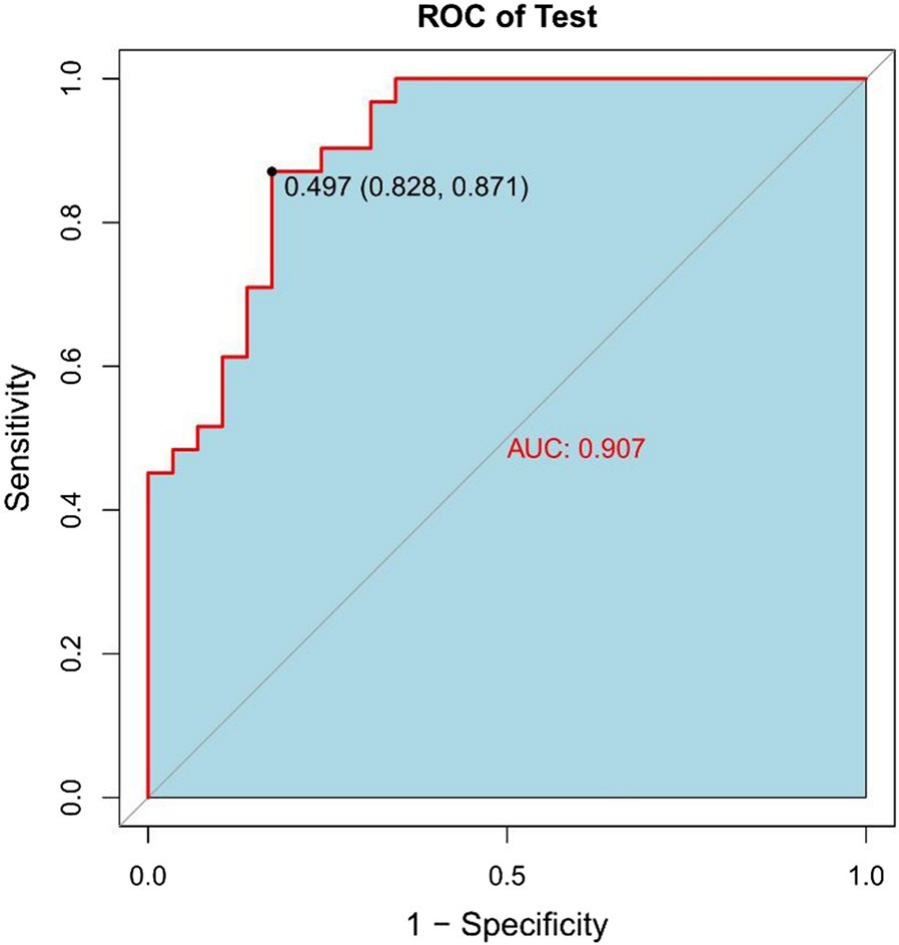
The ROC curve of the radiomics label predicting the treatment of lumbar disc herniation in the validation group was verified

In the normal distribution test of the Rad-score, the P values of both the training group (W = 0.99405, p value = 0.8323) and the validation group (W = 0.98771, p value = 0.8079) are greater than 0.05, indicating that the Rad-score follows a distribution. The Rad-score in the training group was 0.336 ± 0.656 and − 0.336 ± 0.577 in the surgery and conservative patients, respectively, and the difference was statistically significant (t = − 6.4247, p value = 2.103 × 10^−09^ < 0.05). In the validation group, the values were 0.523 ± 0.546 and − 0.531 ± 0.614 in the patents treated by the surgical and conservative measures, respectively. The difference was statistically significant (t = − 7.0145, p value = 3.252 × 10^−9^ < 0.05). The Rad-scores of each patient in the training group and the validation group are shown in Figs. 4 and 5, respectively. In the training group, the ROC curve AUC of the radiomics label for predicting the treatment of lumbar disc herniation was 0.77 (95% CI: 0.70–0.85), with a sensitivity of 65.2%, a specificity of 67.6%, a positive predictive value of 66.2%, and a negative predictive value of 66.7% (as shown in Fig. 6). In the validation group, the AUC was 0.91 (95% CI: 0.83–0.98), the sensitivity was 87.1%, the specificity was 82.8%, the positive predictive value was 84.4%, the negative predictive value was 85.7%, and the accuracy was 85% (Fig. 7).

As shown in Figs. 4 and 5, the higher the score is, the more inclined the patients are to receive surgical treatment. A score greater than 0 represents surgical treatment, while a score less than 0 represents conservative treatment.

### Construction and validation of the radiomics nomogram

**Fig. 8:**
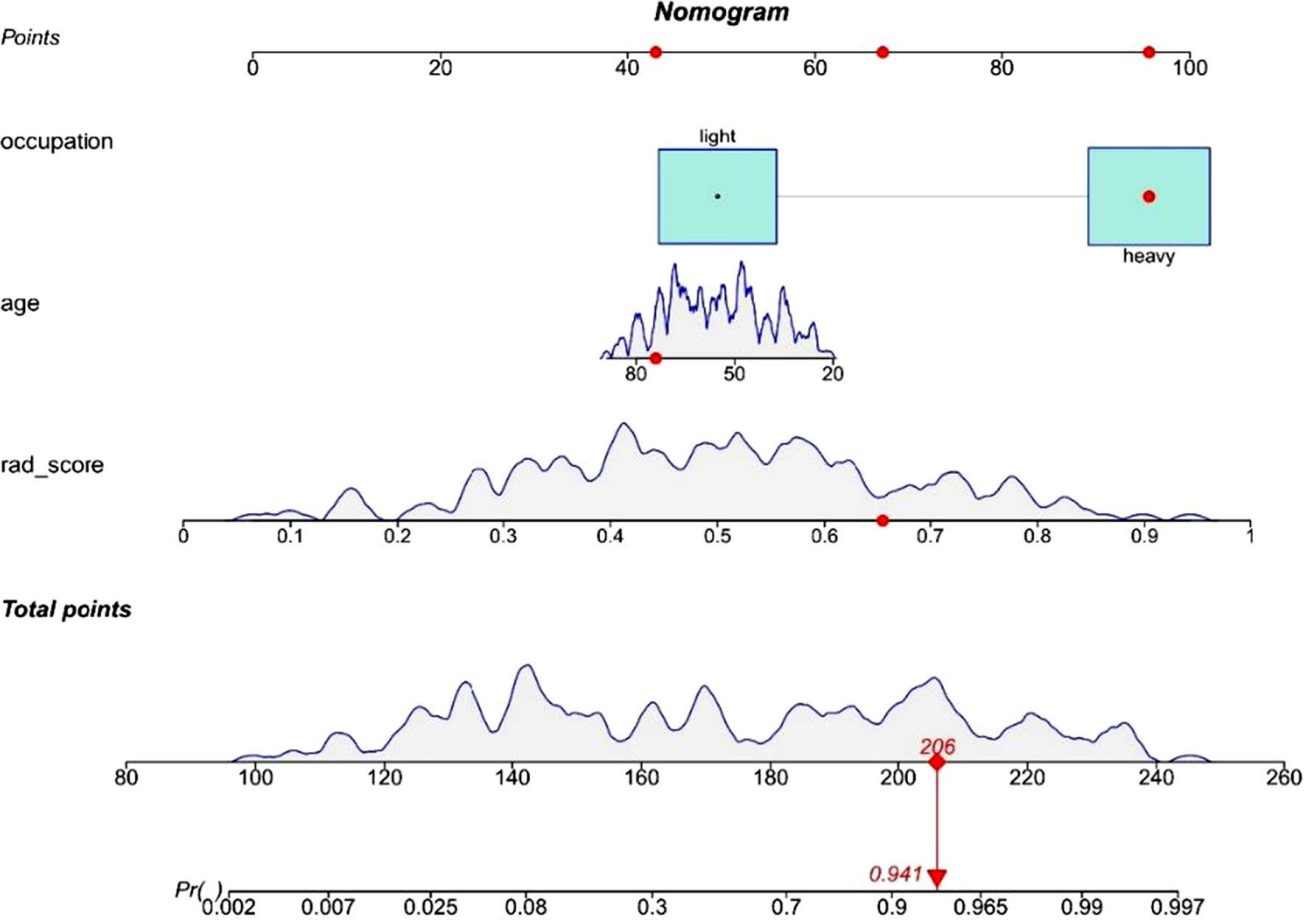
Nomogram of radiomics. The blue line is the distribution trend of radiomics labels, and the red dot is the prediction example of a case

**Fig. 9:**
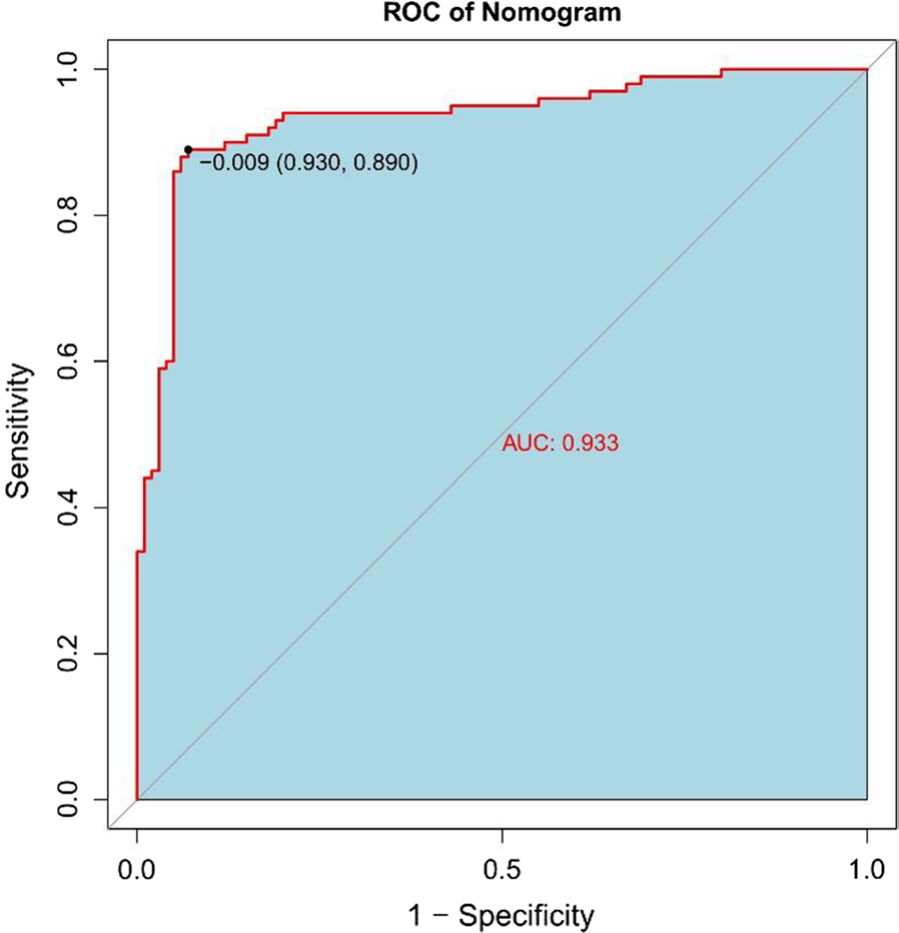
The ROC curve for predicting the treatment of lumbar disc herniation by the radiomics nomogram

**Fig. 10:**
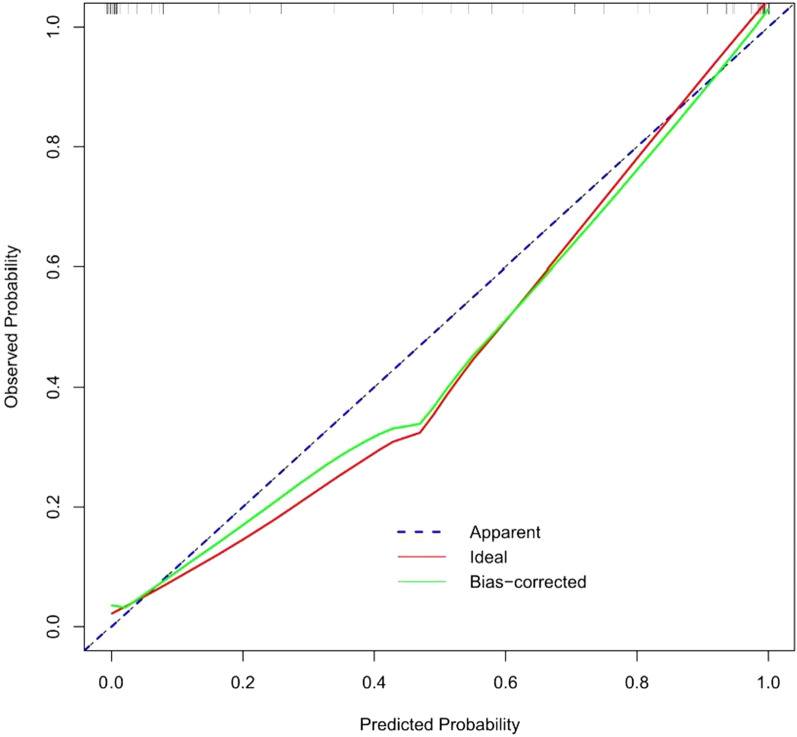
Calibration curve of the radiomics nomogram in the training group

**Fig. 11:**
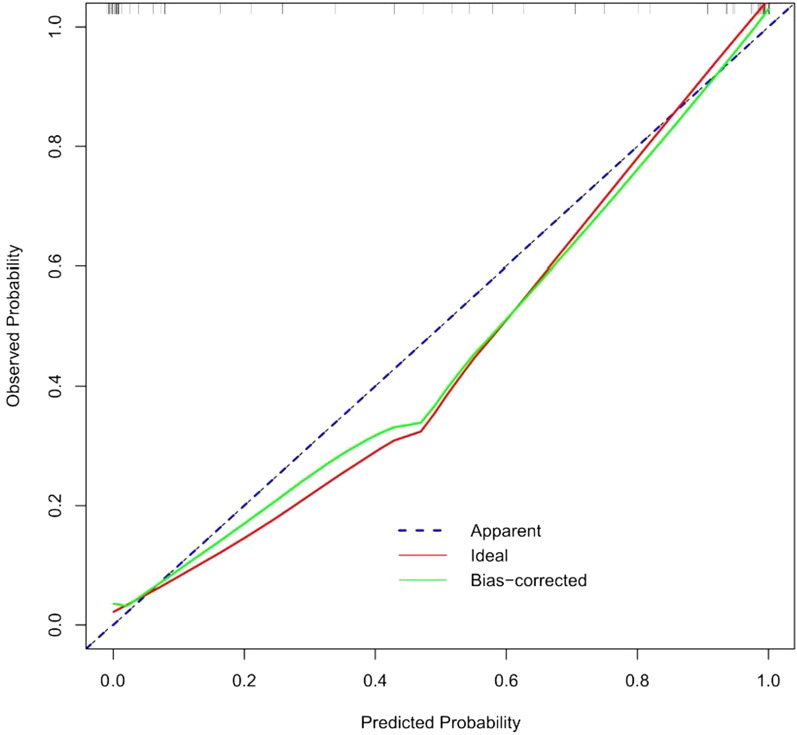
Calibration curve of the radiomics nomogram testing group

**Fig. 12:**
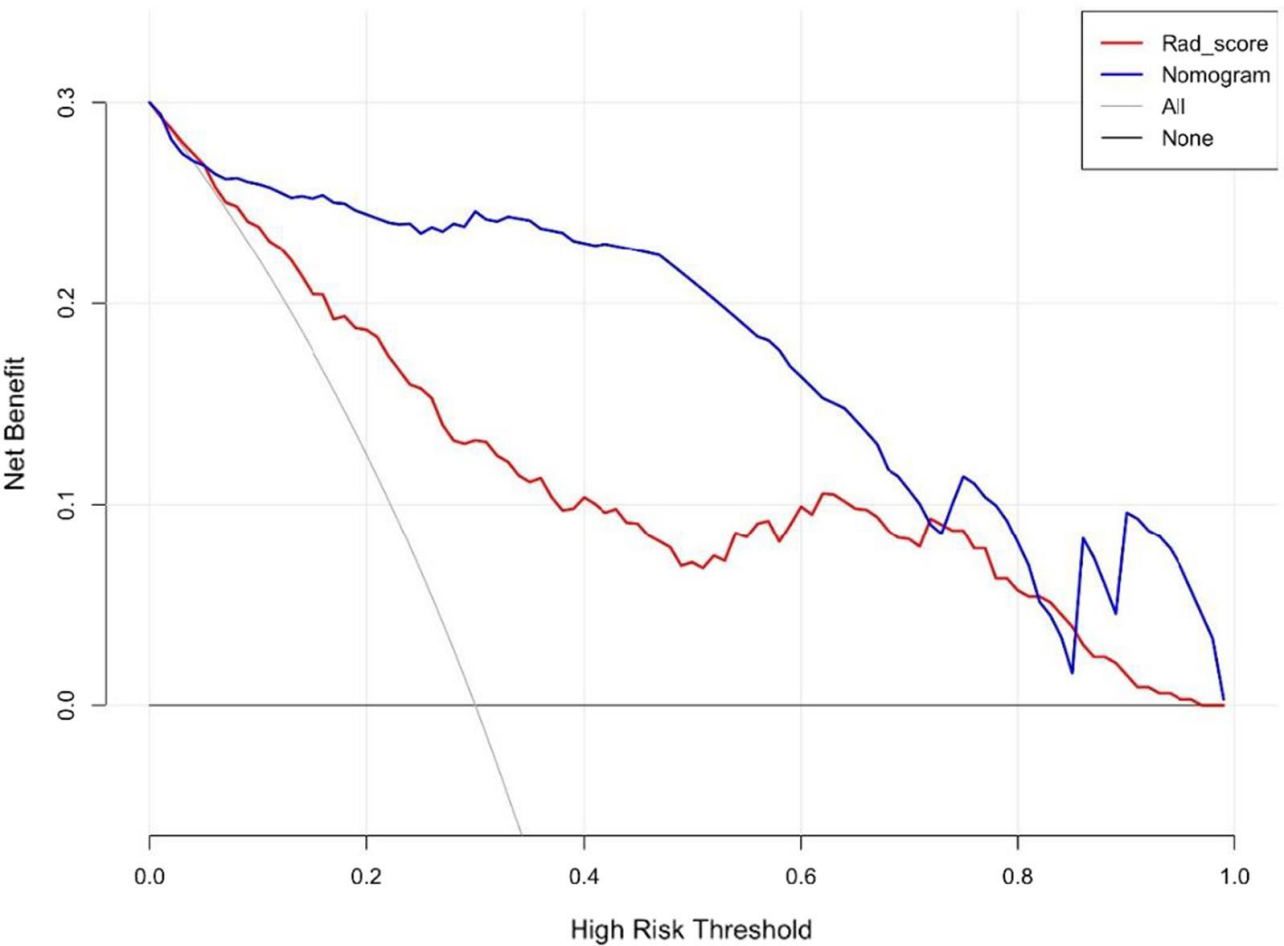
Decision-making curves of the predictive model in all patients with the radiomics label nomogram. The decision curve represents the net benefit under different risk thresholds

Patient information was recorded, and a patient demographic information table was drawn (Table 1). MSU evaluation results were taken as the dependent variable, and high-risk factors were screened out by univariate logistic regression (Table 2) and multivariate logistic regression (Table 3). A nomogram was constructed based on the radiomics label (Fig. 8). The C-index of the radiomics nomogram delivers the treatment plan that has an AUC value of 0.93 (95% CI: 0.90–0.97), a sensitivity of 89%, a specificity of 93%, a positive predictive value of 92.7%, a negative predictive value of 89.4%, and an accuracy of 91% (as presented Fig. 9) for lumbar disc herniation. The correction curves show good agreement between the training group (Fig. 10) and the validation group (Fig. 11) between the prediction and the selection of the actual treatment regimen in the clinic. The Hosmer–Lemeshow test showed that the difference was not statistically significant (X-squared = 12.171, p value = 0.1437 > 0.05), indicating no deviation from fitting. Through the DeLong test, it can be concluded that there are differences between the image omics label model and the line chart model (Table 4). DCA decision curve analysis (Fig. 12) indicated that when the risk threshold was between 5 and 72%, the use of the radiomics label nomogram to predict the treatment of lumbar disc herniation increased the net benefit more than that when using all surgical treatments and all conservative treatments.

**Table 1:** Patient demographic information

	Overall	Conservative	Surgery	p
	n = 200	n = 100	n = 100	
Sex = Female (%)	102 (51.0)	49 (49.0)	53 (53.0)	0.671
Age (Mean (SD))	55.2 (15.7)	54.8 (15.4)	55.5 (16.1)	0.753
Occupation = heavy (%)	103 (51.5)	17 (17.0)	86 (86.0)	< 0.001
Family history = yes (%)	12 (6.0)	7 (7.0)	5 (5.0)	0.767
Bed characteristics = hard (%)	90 (45.0)	43 (43.0)	47 (47.0)	0.67
Smoking = yes (%)	52 (26.0)	28 (28.0)	24 (24.0)	0.629
Physical excrcisc = yes (%)	56 (28.0)	24 (24.0)	32 (32.0)	0.27
BMI (Median [IQR])	21.8[18.7,24.1]	20.8[18.6,22.8]	23.0 [18.8,25.1]	< 0.001

**Table 2:** Univariate logistic regression of treatment style and occupational factors

	OR	95%CI		p value
Occupation. heavy	29.99	14.35	67.18	< 0.001

**Table 3:** Multivariate logistic regression

	OR	95% CI		p value
Sex. female	3.96	1.3	12.76	0.02
Age	0.97	0.94	0.99	0.02
BMI	1.29	1.11	1.52	0.001
Occupation. heavy	46.68	18.62	136.58	< 0.001
Family history. no	0.44	0.06	3.45	0.42
Bed characteristics. hard	1.98	0.83	4.93	0.13
Smoking. no	0.86	0.3	2.39	0.78
Physical exercise. no	2.15	0.85	5.68	0.11

**Table 4:** ROC comparison of the two models (DeLong test)

	ROC comparison of the two models (Delong test)	
rad-score	roc-rad-score-train	roc-rad-score-test
nomogram	roc-nomogram-train	roc-nomogram-test
p-value	< 0.001	0.03

In Figs. 10 and 11, the blue curve represents the ideal prediction performance, the red line represents the actual prediction performance, and the green line represents the corrected prediction performance. The prediction accuracy is higher when the distance between the lines is smaller. As seen from the figures, there is good agreement between the treatment plans predicted by the nomogram and the actual clinician's choice of treatment for lumbar disc herniation.

As seen from the figure, when the risk threshold is between 5 and 72%, the nomogram approach is superior to the rad-score and treats all patients as surgical or conservative.

## Discussion

In this study, we have developed and validated a nomogram that consists of a radiomics label (Rad-score) of lumbar MRI for predicting the treatment of lumbar disc herniation. This nomogram has shown good predictive performance as confirmed by the prediction plan with an AUC of 0.93 (95% CI: 0.90–0.97), a sensitivity of 89%, a specificity of 93%, a positive predictive value of 92.7%, a negative predictive value of 89.4%, and an accuracy of 91%. Our results suggest that the radiomics label nomogram based on lumbar MRI can be used as a quantitative predictive tool to provide clinicians with a reference for treatment choice.

In the past, researchers have tried to combine multidimensional biomarkers with the great potential clinical value identified through high-throughput techniques to model diseases to obtain satisfactory results. The biomarkers included in the model cover different biological scales from molecular to phenotypic [15]. Radiomics in the application of skeletal muscle system is usually in terms of bone tumours, such as bone disease diagnosis and differential diagnosis of tumour prediction of tumour complications, the prognosis of tumour treatment pathologic grading [16–19] and tumour [20], a small study applies beside the osteoporosis [21], Alzheimer's disease [22], temporo-mandibular joint osteoarthritis [23], postoperative infection and inflammation [24], and so on. Few radiomics studies have been conducted on LHD. The Schulthess Klinik orthopaedic team in Zurich, Switzerland, has developed a clinical prognostic model tool [25] that can predict the surgical outcome of disc herniation. The model can help doctors provide patients with truthful information and reasonable expectations for the surgical outcome. In China, there are few similar studies that basically construct diagnostic models related to lumbar disc herniation.

In this study, 11 radiomics features were screened out: the first-order grey nomogram feature (n = 5), the grey level cooccurrence matrix GLCM (n = 4), the grey level correlation matrix GLDM (n = 1), and the grey size region matrix GLSZM (n = 1). Except for the 5 features of the first-order grey nomogram, the other 6 features belong to higher order features of the spatial distribution of the pixel points, which indicates that the first-order 2D and 3D features visible to the naked eye are not enough for the image description of lumbar disc herniation, and they need to be combined with the high-dimensional features that cannot be recognized by the naked eye. Therefore, the 11 quantitative radiomics features included in this study can reflect deeper information of the images of lumbar disc herniation from different perspectives.

In this study, we first investigated the role of an MRI-based radiomics label (Rad-score) in predicting treatment options for lumbar disc herniation. The results indicate that in the training group, the ROC curve AUC of the radiomics label for predicting the treatment of lumbar disc herniation was 0.77 (95% CI: 0.70–0.85), the sensitivity and specificity were 65.2% and 67.6%, the positive predictive value was 66.2%, and the negative predictive value was 66.7%, respectively. In the validation group, the AUC was 0.91 (95% CI: 0.83–0.98), the sensitivity was 87.1%, the specificity was 82.8%, the positive predictive value was 84.4%, the negative predictive value was 85.7%, and the accuracy was 85%. The score magnitude positively related to the inclination of surgical treatment. For instance, a score greater than 0 represented surgical treatment, and a score less than 0 represented conservative treatment. We then constructed the nomogram through the radiomics label, with an AUC of 0.93 (95% CI: 0.90–0.97), a sensitivity of 89%, a specificity of 93%, a positive predictive value of 92.71%, a negative predictive value of 89.42%, and an accuracy of 91%, showing good discriminant efficiency. At the same time, we corrected the nomogram and found that there was good agreement between the treatment plan predicted by the nomogram and the actual clinician's choice of the treatment plan for lumbar disc herniation. The advantage of the clinical application of a nomogram is that it can directly use the graph to calculate the value of a variable. However, discrimination efficiency and calibration may not achieve a specific level of discrimination or the clinical consequences of the degree of miscalibration [26, 27]. To address this problem, clinical decision curve analysis (DCA) was used to evaluate the clinical application of the nomogram. This new strategy provides a net benefit from an in-depth understanding of clinical outcomes based on threshold probability [28, 29]. In this study, the DCA indicated that when the risk threshold was greater than 5%, the nomogram approach was superior to the rad-score and treated all patients as surgical or conservative.

Studies have shown that age, occupation and other factors are significantly related to lumbar disc disease [30–32]. We divide occupation into two grades: light and heavy. If the following conditions are met, occupation will be regarded as heavy [30]: (a) back-loading lifting work involving lifting/upwards pulling of heavy objects and many tonnes of lifting per day for a considerable number of years; (b) back-loading lifting work with generally occurring, extremely heavy and awkward single lifts and several tonnes of lifting per day for a considerable number of years; (c) back-loading care work with many daily handlings of adults or older handicapped children for a considerable number of years; and (d) back-loading, daily exposure to whole-body vibrations from heavily vibrating vehicles for a considerable number of years. The proven effective MSU [33] classification was used in the selection of actual treatment methods. The measurement method is taken from cross-sectional T_2_WI, which considers not only the size of intervertebral disc herniation but also its position under various constraints of local anatomical structure. Applying MSU classification combined with clinical-related factors and symptoms to comprehensively select appropriate treatment methods can effectively reduce the influence of subjective factors.

There were some limitations in this study. First, this was a retrospective study conducted in a single centre with a relatively small sample size. Therefore, a multicentre validation is needed to obtain strong evidence for its clinical application. Second, only one sequence of sagittal T_2_WI was used in this study to extract radiomics features, and current studies have shown that multiparameter MRI sequences can provide more information about lesions [34]. Third, considering that patients may be involved in single or multiple lesions, including clinical factors of patients who may lead to selection bias, only radiomics features were selected for the construction of the nomogram model, since the object of this study was lumbar lesions.

## Conclusion

The nomogram based on a set of radiomics labels for the treatment of lumbar disc prolapse with appropriate predictive values is capable of providing reliable support for the clinical decision-making process and helping clinicians plan surgical strategies and conservative treatment. In addition, nomograms have the capability to provide conservative treatment efficacy in quantitatively providing a reference for clinicians to avoid inadequate and excessive treatments.

## Data Availability

The datasets used and/or analysed during the current study are available from the corresponding author on reasonable request.

## References

[1] Parker SL, Mendenhall SK, Godil SS (2015). Incidence of low back pain after lumbar discectomy for herniated disc and its effect on patient-reported outcomes. Clin Orthop Relat Res.

[2] Chen BL, Guo JB, Zhang HW (2018). Surgical versus non-operative treatment for lumbar disc herniation: a systematic review and meta-analysis. Clin Rehabil.

[3] Zhang B, Xu H, Wang J (2017). A narrative review of non-operative treatment, especially traditional Chinese medicine therapy, for lumbar intervertebral disc herniation. Biosci Trends.

[4] Ma Z, Yu P, Jiang H (2021). Conservative treatment for giant lumbar disc herniation: clinical study in 409 cases. Pain Physician.

[5] Lurie JD, Tosteson TD, Tosteson AN (2014). Surgical versus nonoperative treatment for lumbar disc herniation: eight-year results for the spine patient outcomes research trial. Spine (Phila Pa 1976).

[6] Postacchini F (1996). Results of surgery compared with conservative management for lumbar disc herniations. Spine.

[7] Gillies RJ, Kinahan PE, Hricak H (2016). Radiomics: images are more than pictures, they are data. Radiology.

[8] Yushkevich PA, Piven J, Hazlett HC (2006). User-guided 3D active contour segmentation of anatomical structures: significantly improved efficiency and reliability. Neuroimage.

[9] Huang YQ, Liang CH, He L (2016). Development and validation of a radiomics nomogram for preoperative prediction of lymph node metastasis in colorectal cancer. J Clin Oncol.

[10] Hoo ZH, Candlish J, Teare D (2017). What is an ROC curve?. Emerg Med J.

[11] Kamarudin AN, Cox T, Kolamunnage-Dona R (2017). Time-dependent ROC curve analysis in medical research: current methods and applications. BMC Med Res Methodol.

[12] Kramer AA, Zimmerman JE (2007). Assessing the calibration of mortality benchmarks in critical care: the Hosmer-Lemeshow test revisited. Crit Care Med.

[13] Wolbers M, Koller MT, Witteman JC (2009). Prognostic models with competing risks: methods and application to coronary risk prediction. Epidemiology.

[14] Vickers AJ, Elkin EB (2006). Decision curve analysis: a novel method for evaluating prediction models. Med Decis Making.

[15] Younesi E, Hofmann-Apitius M (2013). From integrative disease modeling to predictive, preventive, personalized and participatory (P4) medicine. EPMA J.

[16] Haimei C, Jin L, Zixuan C (2020). Value of radiomics nomogram based on T1WI for pretreatment prediction of relapse within 1 year in osteosarcoma: a multicenter study. Chin J Radiol.

[17] Wang H, Chen H, Duan S (2020). Radiomics and machine learning with multiparametric preoperative MRI may accurately predict the histopathological grades of soft tissue sarcomas. J Magn Reson Imaging.

[18] Zhang J, Sun J, Han T (2020). Radiomic features of magnetic resonance images as novel preoperative predictive factors of bone invasion in meningiomas. Eur J Radiol.

[19] Pan J, Zhang K, Le H (2021). Radiomics nomograms based on non-enhanced MRI and clinical risk factors for the differentiation of chondrosarcoma from enchondroma. J Magn Reson Imaging.

[20] Liu Q, Li J, Liu F (2020). A radiomics nomogram for the prediction of overall survival in patients with hepatocellular carcinoma after hepatectomy. Cancer Imaging.

[21] Rastegar S, Vaziri M, Qasempour Y (2020). Radiomics for classification of bone mineral loss: a machine learning study. Diagn Interv Imaging.

[22] Tang L, Wu X, Liu H (2021). Individualized prediction of early Alzheimer's disease based on magnetic resonance imaging radiomics, clinical, and laboratory examinations: a 60-month follow-up study. J Magn Reson Imaging.

[23] Bianchi J, de Oliveira RA, Gonçalves JR (2020). Osteoarthritis of the Temporomandibular Joint can be diagnosed earlier using biomarkers and machine learning. Sci Rep.

[24] D'amico N, Gandolfo P, Valbusa G (2018). A radiomic approach for successful distinction of infection versus infammation in patients treated with reparative orthopaedic surgery: a pilot study. Eur J Nucl Med Mol Imaging.

[25] Staub LP, Aghayev E, Skrivankova V (2020). Development and temporal validation of a prognostic model for 1-year clinical outcome after decompression surgery for lumbar disc herniation. Eur Spine J.

[26] Localio AR, Goodman S (2012). Beyond the usual prediction accuracy metrics: reporting results for clinical decision making. Ann Intern Med.

[27] Van Calster B, Vickers AJ (2015). Calibration of risk prediction models: impact on decision-analytic performance. Med Decis Making.

[28] Wu S, Zheng J, Li Y (2017). A radiomics nomogram for the preoperative prediction of lymph node metastasis in bladder cancer. Clin Cancer Res.

[29] Zhang L, Dong D, Li H (2019). Development and validation of a magnetic resonance imaging-based model for the prediction of distant metastasis before initial treatment of nasopharyngeal carcinoma: a retrospective cohort study. EBioMedicine.

[30] Schumann B, Bolm-Audorff U, Bergmann A (2010). Lifestyle factors and lumbar disc disease: results of a German multi-center case-control study (EPILIFT). Arthritis Res Ther.

[31] Pye SR, Reid DM, Adams JE (2007). Influence of weight, body mass index and lifestyle factors on radiographic features of lumbar disc degeneration. Ann Rheum Dis.

[32] Seidler A, Bergmann A, Jäger M (2009). Cumulative occupational lumbar load and lumbar disc disease–results of a German multi-center case-control study (EPILIFT). BMC Musculoskelet Disord.

[33] Mysliwiec LW, Cholewicki J, Winkelpleck MD (2010). MSU classification for herniated lumbar discs on MRI: toward developing objective criteria for surgical selection. Eur Spine J.

[34] Liu Z, Li Z, Qu J (2019). Radiomics of multiparametric MRI for pretreatment prediction of pathologic complete response to neoadjuvant chemotherapy in breast cancer: a multicenter study. Clin Cancer Res.

